# Scale-integrated Network Hubs of the White Matter Structural Network

**DOI:** 10.1038/s41598-017-02342-7

**Published:** 2017-05-26

**Authors:** Hunki Kwon, Yong-Ho Choi, Sang Won Seo, Jong-Min Lee

**Affiliations:** 1 Department of Biomedical Engineering, Hanyang University, Seoul, South Korea; 2 Department of Neurology, Samsung Medical Center, Sungkyunkwan University School of Medicine, Seoul, South Korea

## Abstract

The ‘human connectome’ concept has been proposed to significantly increase our understanding of how functional brain states emerge from their underlying structural substrates. Especially, the network hub has been considered one of the most important topological properties to interpret a network as a complex system. However, previous structural brain connectome studies have reported network hub regions based on various nodal resolutions. We hypothesized that brain network hubs should be determined considering various nodal scales in a certain range. We tested our hypothesis using the hub strength determined by the mean of the “hubness” values over a range of nodal scales. Some regions of the precuneus, superior occipital gyrus, and superior parietal gyrus in a bilaterally symmetric fashion had a relatively higher level of hub strength than other regions. These regions had a tendency of increasing contributions to local efficiency than other regions. We proposed a methodological framework to detect network hubs considering various nodal scales in a certain range. This framework might provide a benefit in the detection of important brain regions in the network.

## Introduction

The ‘human connectome’ concept, which refers to a comprehensive structural description of the network of elements and connections forming the human brain, has been proposed to significantly increase our understanding of how functional brain states emerge from their underlying structural substrates^1, 2^. While neurons are arranged in an unknown number of anatomically distinct regions and areas in the human cerebral cortex^3^, anatomically distinct brain regions and inter-regional pathways represent perhaps the most feasible organizational level for the human connectome^1, 2^. Graph theoretical analysis, a powerful way of quantifying topological properties of a network, has been adopted in neuroimaging studies to investigate the characteristics of human brain networks on a macroscale^4–8^. This makes it possible to consider the human brain as a complex system, where nodes are the regions of the brain and edges represent the interacting between them. It means that the regions of the human brain affect each other rather than working independently. For the construction of the structural network of the human brain, macroscopic gray matter (GM) regions are defined as nodes and the fibers connecting them are defined as edges in neuroimaging data^2, 9–12^. Diffusion tensor imaging (DTI), which can measure the structural integrity of white matter (WM) fiber tracts, is one of the most important imaging modalities for understanding the structural connectivity between brain regions^13, 14^. DTI also gives more information on the pathophysiological procedures than other imaging modalities in terms of brain network analysis^4, 8, 15–17^.

Many network parameters, which include global network topological properties such as clustering coefficients, global efficiencies, path length, small-worldness and local network topological properties such as nodal degree, betweenness centrality, and local efficiencies, have been suggested to explain the topology of the network^2, 18^. Especially, the hub, which plays a key role in efficient communication in a network, has been considered one of the most important topological properties to interpret a network as a complex system^19, 20^. A network hub is generally defined as the nodes of network with high degrees or high centrality and is determined based on how many of the minimum paths between all other node pairs in the network pass through it^1^. For example, a network hub of power grids makes it possible to easily distribute the load of one station to other stations, reducing the risk of serious failure^21, 22^. A network hub has been considered important in the study of how a disease spreads in a network because the loss of a hub is likely to break a network into disconnected parts^23^.

Previous structural brain connectome studies have reported network hub regions based on various nodal resolutions^4, 24–27^. Many studies have used a predefined atlas such as the 90 cortical regions from the Automated Anatomical Labeling atlas (AAL)^28^ to define a network node^4, 29^. On the contrary, Nijhuis *et al*. used 500 random regions as nodes to define a network hub^30^. Hagman *et al*. used 998 regions that equally cover the whole brain to define the network nodes and show the network hub regions^17^. Zalesky *et al*. reported brain network analysis results across random subdivisions at scales from 100 to 4000 on the AAL template^24^. Romero-Garcia *et al*. also used different cortical sub-regions from 66 to 1494 on the Desikan–Killiany atlas for network analysis^26^. Supplementary Table S1 summarized the detailed hub regions of these studies. Interestingly, these studies reported different hub regions, which might be due to the strong dependence of network hub regions on the network nodal scale^24^. The strength of a regional connection was found to positively correlate with the size of the region’s surface in tract-tracing studies of macaque monkeys^31^. The strength of this relationship between region size and network hub regions might be associated with the number of tracts measured in a given hub region depending on the size of the particular region^25^. Since most previous studies have defined network hubs after restriction to a specific resolution, they might not capture the potential scale-dependent nature of a node’s role in the network as a whole^32^. Some studies have shown the strong dependence between hub regions and the network nodal scale, but did not calculate the dependence quantitatively^24, 26^.

We hypothesized that brain network hubs should be determined considering various nodal scales in a certain range. To the best of our knowledge, the effects of nodal scale on the network hub regions and which regions are scale-integrated hubs (H_IS_) considering the nodal scale changes have not been examined. We tested our hypothesis using the hub strength determined by the mean of the “hubness” values over a range of nodal scales. We normalized the group hub maps of each nodal scale allowing an unbiased comparison between the hub values of multiple nodal scales and defined the H_IS_ on the hub strength map using z-score transformation.

## Results

### Ratio of short association fibers

The wiring patterns of WM fibers directly define the topological performance of brain networks. We computed the ratio of the number of connections of short association fibers (short fibers and U-fibers) to the maximum possible number of connections at each nodal scale to understand how the number of short fibers and U-fibers affect network hubs in different nodal scales (Table 1). The short fibers and U-fibers were defined as the edges but not included in calculation of the network hubs. The ratio showed that the number of short fibers and U-fibers steadily decreased as the number of nodal scales increased.

**Table 1:** The ratio of the number of short fibers and U-fibers to the maximum possible number of connections at each nodal scale.

#Nodal scale	Mean (%)	Std (%)
100	0.1866	0.0237
200	0.0942	0.0156
300	0.0599	0.0114
400	0.0409	0.0079
500	0.0300	0.0063
600	0.0230	0.0052

The number of short fibers and U-fibers were determined based on the edges not included in calculation of the network hubs. The ratio showed that the number of short fibers and U-fibers steadily decreased as the nodal scale increased.

### Network hub of multiple scales

**Figure 1:**
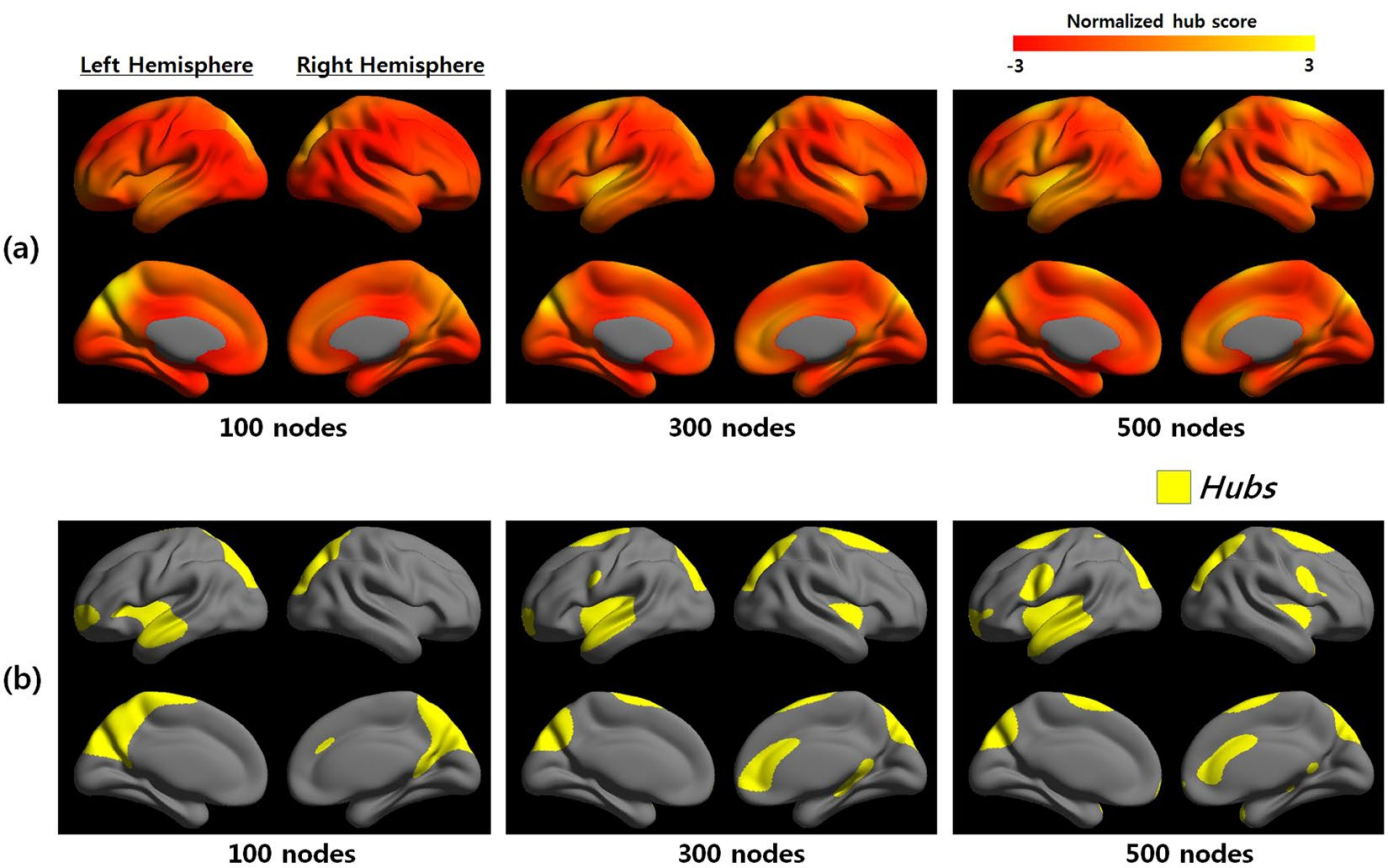
Betweenness centrality maps and hubs at multiple scales. (**a**) Betweenness centrality maps were shown at three network nodal scales (100, 300, and 500). The colored scale represented the hub score (red to yellow). (**b**) Hubs of three network nodal scales were defined by a standard deviation greater than the mean of the betweenness centrality hub map. Supplementary Table S3 showed the network hub at each scale in detail.

Figure 1 showed the average group hub map at each nodal scale. Betweenness centrality maps at different nodal scales were presented, where regions of yellow color indicate higher hub scores than regions of red color (Fig. 1, top). We identified group hubs according to the average betweenness centrality map that were one standard deviation above the mean (see Methods). Hubs of networks at multiple scales were highlighted in yellow (Fig. 1, bottom). The anatomical location of the hubs can be represented from the pre-defined template^28^ and abbreviations for the cortical regions were listed in Supplementary Table S2. Note that most of the hub regions almost overlapped, among which six cortical regions (precuneus, cuneus, superior temporal gyrus, superior frontal gyrus, superior parietal gyrus, and superior occipital gyrus) appeared as hubs in a bilaterally symmetric fashion between nodal scales. In addition, four brain regions (right middle occipital gyrus, right inferior frontal gyrus, left inferior frontal gyrus, and left Heschl’s gyrus) were considered as hubs only for the coarsest scale; whereas, three different brain regions (right anterior cingulate and paracingulate gyri, left precentral gyrus, and right precentral gyrus) were identified as hubs only in the finest scale. Supplementary Table S3 showed the anatomical locations of the hubs at multiple scales (100–600 scales) and ratio of each hub region compared with pre-defined template.

### H_IS_ region distribution

**Figure 2:**
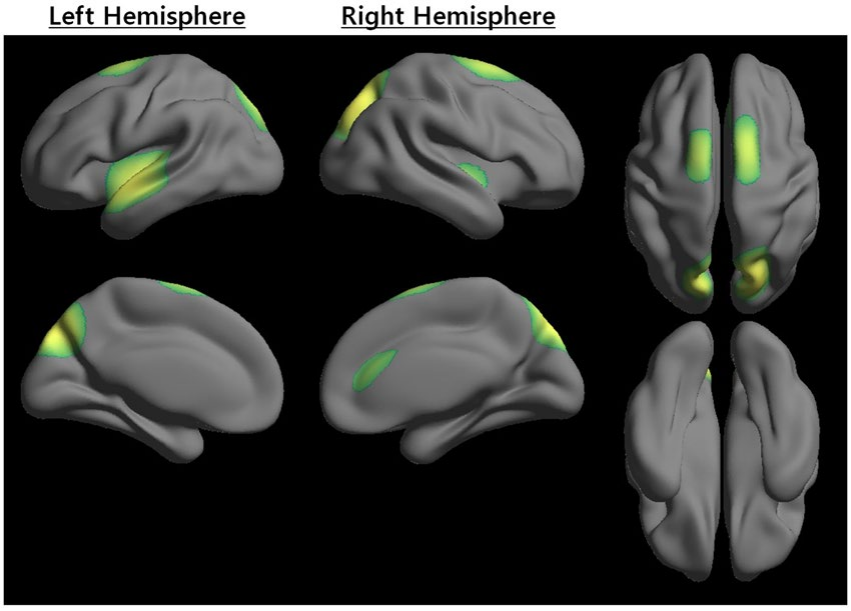
Distribution of scale-integrated hub (*H*_*IS*_). Z-score transformation was performed to combine the group hub scores from the different scales. Note that the scale-integrated hub score (*H*_*IS*_ *_*_*SC*_) captured how ‘well connected’ node i was to other nodes in the scale-integrated hub strength (*H*_*IS*_ *_*_*ST*_) map. Regions with a high *H*_*IS*_ *_*_*SC*_ might also indicate a fundamental hub with high consistency across the nodal scale. Note that Table 2 showed the *H*_*IS*_ regions in detail.

The *H*_*IS*_ *_*_*ST*_ map was shown to demonstrate the overall network hub pattern between multiple nodal scales (Supplementary Figure S1). Figure 2 showed *H*_*IS*_ regions with *H*_*IS*_ *_*_*SC*_ greater than one standard deviation above the mean of the *H*_*IS*_ *_*_*ST*_ map. Note that, as shown in Table 2, *H*_*IS*_ included the regions precuneus, superior occipital gyrus, superior parietal gyrus, insula, superior temporal gyrus in a bilaterally symmetric fashion, and right cingulum. Additionally, some regions of the precuneus, superior occipital gyrus, and superior parietal gyrus in a bilaterally symmetric fashion had a relatively higher level of *H*_*IS*_ *_*_*SC*_ than other regions of the insula, superior temporal gyrus in a bilaterally symmetric fashion, and right cingulum.

**Table 2:** Scale-integrated hubs (*H*_*IS*_) (scale-integrated hub score (*H*_*IS*_ *_*_*SC*_) >1).

Anatomical location	*H*_*IS*_ *_*_*ST*_	*H*_*IS*_ *_*_*SC*_
Right superior occipital gyrus	0.980028	4.507347
Left superior occipital gyrus	0.898464	4.095554
Right precuneus	0.948762	4.349493
Left precuneus	0.920223	4.20541
Right insula	0.611848	2.648516
Left insula	0.894113	4.073586
Right superior temporal gyrus	0.615138	2.665126
Left superior temporal gyrus	0.961131	4.41194
Right superior parietal gyrus	0.980018	4.507295
Left superior parietal gyrus	0.878207	3.993282
Right cingulum	0.723567	3.124552

Anatomical locations of the top 11 *H*_*IS*_ that had an *H*_*IS*_ *_*_*SC*_ one standard deviation above the mean.

### Validation of H_IS_

**Figure 3:**
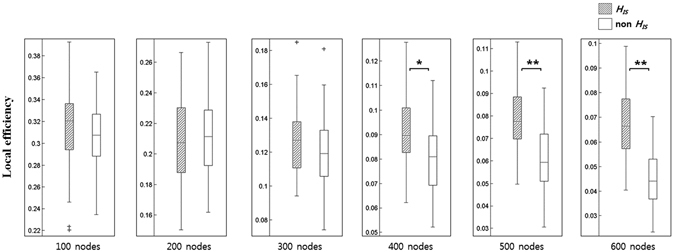
Validation of scale-integrated hub (*H*_*IS*_). The *H*_*IS*_ regions had a tendency of increasing contributions to local efficiency than the non *H*_*IS*_ regions at most scales. The error bars represented the standard deviation. Note that ** and * designated statistically significant differences with p < 0.001 and p < 0.05, respectively.

We validated whether *H*_*IS*_ regions involved a high level of local efficiency by comparing non *H*_*IS*_ regions at each nodal scale in order to demonstrate the importance of the *H*_*IS*_ regions. The *H*_*IS*_ regions had a tendency of increasing contributions to local efficiency than the non *H*_*IS*_ regions at most scales (Fig. 3). The p-values, obtained by performing a two-sample t-test for each scale, were also provided to indicate the significance of difference in *H*_*IS*_ and non *H*_*IS*_ regions. The local efficiency in *H*_*IS*_ regions was significantly higher than that of non *H*_*IS*_ regions at 400 nodes (p < 0.05, t-test) or more (p < 0.001, t-test). The Kolmogorov–Smirnov test showed that the distribution of local efficiency in *H*_*IS*_ and non *H*_*IS*_ regions for each of the nodal scales met assumptions of normality (p > 0.05).

## Discussion

We proposed a methodological framework to detect network hubs considering various nodal scales in a certain range in this work. Our results suggested that the mechanism for forming structural network hubs is a scale-dependent process and structural network hubs should be determined by investigating the trade-off between cortical scales. This framework could provide biologically meaningful results and reduce the bias against network scales by considering all levels of network hubs instead of restricting to one nodal scale. Contrary to previous studies that evaluated network topology at a single nodal network^4, 17, 29, 33^, our results highlighted the importance of scale integration to detect fundamental brain network topological properties by using *H*_*IS*_ *_*_*ST*_. It demonstrated the performance of the suggested method for different numbers of nodes between 100 and 600 while avoiding any arbitrary choices.

Some studies have also proposed network hub analysis of multiple nodal scales. Zalesky *et al*. reported that the cingulate was detected as a region that is highly connected with other regions in the coarsest scale, while the anterior cingulate was detected in only the finest scale^24^. Romero-Garcia *et al*. demonstrated that some regions of the middle frontal gyrus, middle occipital gyrus, cuneus, and precuneus were detected in the coarsest scale, while almost all frontal regions, inferior occipital gyrus, inferior parietal gyrus, and precuneus were detected in only the finest scale^26^. These studies showed that there was a strong dependence between hub region and network nodal scale; however, they did not calculate the dependence quantitatively considering the nodal scale changes.

Some regions of the middle occipital gyrus and inferior frontal gyrus were defined as network hubs at the coarsest scale, but regional heterogeneity was reduced at the finest scale in our results. Other regions of the cingulate and precentral gyrus were defined as network hubs in the finest scale. While all of these regions have been reported as network hubs in previous studies^9, 29^, they were variable according to the nodal scales.

Our findings showed that *H*_*IS*_ regions considering all scales were identified in the precuneus, superior occipital gyrus, and superior parietal gyrus regions. The precuneus has mutual corticocortical connections with neighboring areas that are responsible for the anatomical basis of their functional coupling and is also connected with other parietal areas related to visuo-spatial information processing^34^. The precuneus plays a core role in the brain network, suggesting that it has an important function relative to the other regions^35^. The *H*_*IS*_ regions in the precuneus, superior occipital gyrus, and superior parietal gyrus had a significant negative correlation between age and regional efficiency in a bilaterally symmetric fashion^16^. Importantly, these *H*_*IS*_ regions have also been consistently observed as network hubs^9, 29^.

It was shown that the *H*_*IS*_ regions had significantly greater contributions to local efficiency than the non *H*_*IS*_ regions at some nodal scales (400,500 and 600). Note that the *H*_*IS*_ regions had a tendency of increasing contributions to local efficiency than the non *H*_*IS*_ regions at most scales except for one scale (200). Since network hubs tend to have a high level of local efficiency^36^, the *H*_*IS*_ regions might play an important role in the network and can be considered as fundamental regions, that is, hubs. Although hubs were defined at each scale, our framework can separate them into the *H*_*IS*_ regions and the non *H*_*IS*_ regions, and the *H*_*IS*_ regions had relatively higher betweenness centrality than non *H*_*IS*_ regions (Supplementary Figure S2). Our framework method could achieve an increased sensitivity by eliminating the effect of the specific scale on the overall network hub pattern.

We considered that two regions were connected when three fiber tracts were located in these two regions. We analyzed all networks at their fundamental links since structural network based on DTI is naturally sparse. Spurious connections of two regions could be induced by noises or limitations of deterministic tractography. We showed the number of edges according to a threshold of fibers in the Supplementary Figure S3. Even if the threshold of fibers was changed, the edges were similar according to nodal scales. It also showed that the important connections having large amount of fibers remained unchanged. We, therefore, selected three fibers as threshold, which was commonly adopted in the previous network studies to eliminate these spurious connections.

**Figure 4:**
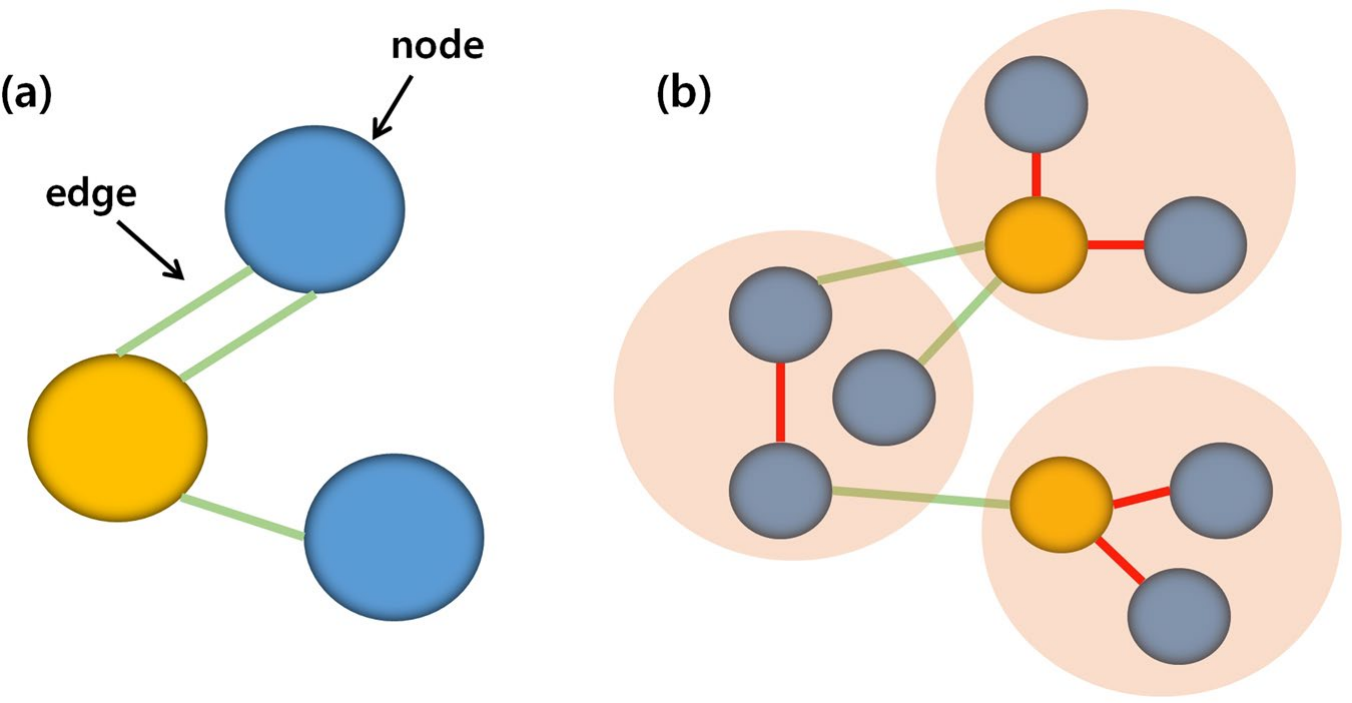
Changes in the network hub position at different scales. A simple graphical model showed a mathematical description of a network, consisting of nodes (blue circles), hubs (yellow circles), connections between the nodes (green lines), and u-fibers or short fibers of nodes (red lines). The schematic diagram represented the different hub regions at different nodal scales.

The number of short association fibers might affect the varied hub pattern according to the nodal scale. Short association fibers mostly include the local associative fibers (U-fibers) and neighborhood association fibers^37^. Short association fibers composed of short fibers and U-fibers can have an effect in network hub regions^38^. Figure 4 showsed an example illustration of how short association fibers can affect hub location according to the nodal scale. Table 1 showed that the number of short association fibers has steadily decreased as the number of nodal scales increased. Previous studies have reported that short association fibers were susceptible to aging effects and were less myelinated, while long association fibers had thicker myelination along the neuron and axon^39^. It suggested that the removal of short association fibers contributes to lower cognitive efficiency and higher compensatory brain activation^40^.

While an *H*_*IS*_ *_*_*SC*_ of 1 was used to define *H*_*IS*_ regions in this study, we validated the various *H*_*IS*_ *_*_*SC*_ (0.5, 1, 1.5, 2) values to see their effects on determining the anatomical hub regions (Supplementary Figure S4). The overall hub distribution showed a similar pattern even if the *H*_*IS*_ *_*_*SC*_ changed, which might imply that *H*_*IS*_ regions have high consistency of forming a network hub. We also used 1 standard deviation in this study to compare and interpret with the existing network hub results^8, 14–25, 29, 41–52^.

We used betweenness centrality to detect network hub regions. Hubs, however, can be detected using other network centrality measures with high degrees, high closeness centrality, and high rich-club properties compared with the rest of the network^20, 53^. Although many studies have used betweenness centrality to examine the regional hub^2, 29^, these various measures of centrality could help interpret the meaning of *H*_*IS*_ regions in the network. We used a uniform upsampling of the whole brain surface without respecting anatomical boundaries, which could take an important role when hub regions are found at the boundaries of the predefined template^30^.

Romero-Garcia *et al*. suggested that the best topological trade-off of network scales is in a range from 540–599 and did not differ much from the finest cortical scale^26^. Nijhuis *et al*. parcelled neocortical regions into 500 ROIs to detect network hubs. Many previous studies also investigated anatomical connections by dividing into around 100 regions. Therefore, we used a range from 100–600 nodal scales to determine *H*_*IS*_ *_*_*SC*_ in this study. Our results demonstrated that the hub regions that were implied by our proposed methodological framework have important roles in the study of brain network analysis.

## Methods

### Overview

**Figure 5:**
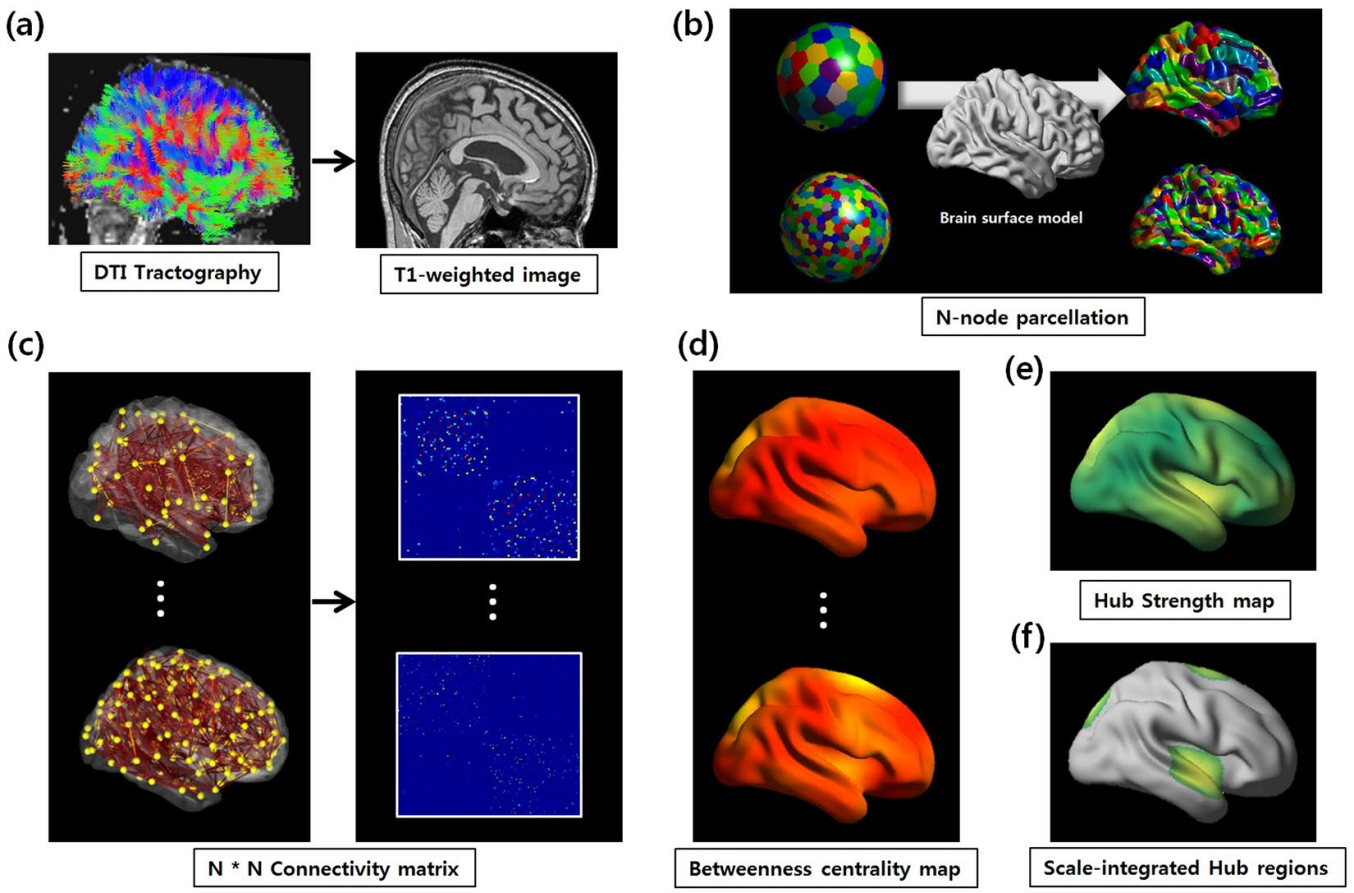
Flowchart of the processing pipeline. (**a**) The T1-weighted image was rigidly coregistered to the averaged b0 image in the native diffusion space. The whole-brain WM tracts were reconstructed using the FACT algorithm. (**b**) Each neocortical hemisphere was parcellated 20 times into 100, 200, 300, 400, 500, or 600 regions of interest (ROIs) as nodes using the k-means algorithm determined with the Euclidean distances between coordinates on the sphere model (left). The parcellated ROIs were transformed to the WM surface matched with the sphere model (right). (**c**) Two nodes were considered to be structurally connected by an edge when at least the end points of three fiber tracts were located in these two regions, and weighted structural networks were constructed for each individual node at each scale. (**d**) The betweenness centrality map was calculated 20 times for each pre-defined individual connectivity matrix dataset using the Brain Connectivity Toolbox (http://www.brain-connectivity-toolbox.net). Individual betweenness centrality maps were averaged to create the group hub map. This procedure was repeated for all nodal scales. (**e**) The scale-integrated hub strength (*H*_*IS*_ *_*_*ST*_) is defined as the sum of all normalized group hub scores divided by the total number of nodal scales in order to estimate the overall network hub pattern between multiple nodal scales. (**f**) The scale-integrated hub score (*H*_*IS*_ *_*_*SC*_) captures how ‘well connected’ node i is to other nodes in the *H*_*IS*_ *_*_*ST*_ map.

The aim of the analysis pipeline presented here was to identify network hub regions considering various nodal scales in a certain range. The flowchart in Fig. 5 described the main steps of our analysis process.

### Subjects and MRI acquisition

The Institutional Review Board (IRB) of Samsung Medical Center approved this study. All participants in this study provided written informed consent. We used a dataset from 54 healthy subjects from Samsung Medical Center, Seoul, Korea. Three-dimensional (3D) T1 turbo field echo magnetic resonance (MR) images were acquired using the same 3.0-T magnetic resonance imaging (MRI) scanner (Philips 3.0T 164 Achieva, Eindhoven, the Netherlands) with the following imaging parameters: sagittal slice thickness, 1.0 mm, over contiguous slices with 50% overlap; no gap; repetition time (TR) of 9.9 msec; echo time (TE) of 4.6 msec; flip angle of 8°; and matrix size of 240 × 240 pixels, reconstructed to 480 × 480 over a field of view of 240 mm. The following parameters were used for the 3D fluid-attenuated inversion recovery (FLAIR) images: axial slice thickness of 2 mm; no gap; TR of 11000 msec; TE of 125 msec; flip angle of 90°; and matrix size of 512 × 512 pixels.

Sets of axial diffusion-weighted single-shot echo-planar images were collected in the whole-brain DT-MRI examination with the following parameters: 128 × 128 acquisition matrix, 1.72 × 1.72 × 2 mm^3^ voxels; reconstructed to 1.72 × 1.72 × 2 mm^3^; 70 axial slices; 22 × 22 cm^2^ field of view; TE 60 ms; TR 7696 ms; flip angle 90°; slice gap 0 mm; and b-factor of 600 smm^−2^. Diffusion-weighted images were acquired from 45 different directions with the baseline image without weighting [0, 0, 0]. All axial sections were acquired parallel to the anterior commissure-posterior commissure line.

### Tissue classification and surface modeling

An automated processing-pipeline (CIVET) was used to extract surfaces of the inner and outer cortex (http://mcin-cnim.ca/neuroimagingtechnologies/civet/)^54^. Surfaces consisting of 41,962 vertices were generated for each hemisphere using deformable spherical mesh models after correction for intensity non-uniformity, normalization to the MNI 152 template, removal of non-brain tissues, and tissue classification of WM, GM, cerebrospinal fluid, and background using an advanced neural-net classifier^54–57^.

### DTI preprocessing

DTI data was processed using the FMRIB Software Library (http://www.fmrib.ox.ac.uk/fsl). Motion artifacts and eddy current distortions were corrected by normalizing each diffusion-weighted volume to the baseline volume (b0) using the affine registration method in the FMRIB’s Linear Image Registration Tool (FLIRT). Diffusion tensor matrices from the sets of diffusion-weighted images were generated using a general linear fitting algorithm.

The DTI tractography was performed using the FACT algorithm^58^ implemented in the Diffusion Toolkit in the diffusion MR space, and about 100,000 fibers were extracted in each subject (Fig. 5(a)). An angle less than 45° between each fiber tracking step and minimum/maximum path length of 20/200 mm were included in a threshold set of the terminating condition. The tractography result was masked by the classified WM map.

### Parcellating the cortex with different resolutions

Each neocortical hemisphere was parcellated into 100, 200, 300, 400, 500, or 600 regions of interest (ROIs) with similar size as nodes instead of pre-defined anatomical template at single scale. The k-means algorithm was used based on the Euclidean distances between coordinates on a sphere model. The sphere model was made by spherical harmonic parameterization and the nodes were sampled in a 3D point distribution model (SPHARM-PDM)^59^. It was repeated 20 times (Fig. 5(b), left) to reduce the biases for random selection of nodes and size of them for each individual at each nodal scale. Twenty random nodes were, then, transformed to a WM surface matched with the sphere model (Fig. 5(b), right).

### Connectivity matrix and group hub map extraction

T1-weighted images were co-registered to the b0 images using FLIRT. Reconstructed whole-brain fiber tracts were inversely transformed into the T1 space, and fiber tracts and surface-based parcellated regions at various scales were located in the same space. Two nodes were considered to be structurally connected by an edge when at least the end points of three fiber tracts were located in these two regions, and the edge was defined by the number of fiber tracts. We selected the threshold of fiber number as three, which was commonly adopted in the previous network studies^4, 60–62^ to eliminate spurious connections. Finally, weighted structural networks were constructed for each individual at each scale (Fig. 5(c)).

Nodal betweenness centrality was adopted to examine the regional hub characteristics of the structural brain networks^2, 29^. The betweenness centrality (BC) of a node i is defined as

1 $${\boldsymbol{BC}}({\boldsymbol{i}})=\sum _{{\boldsymbol{j}}\ne {\boldsymbol{i}}\ne {\boldsymbol{k}}}\frac{{{\boldsymbol{\rho }}}_{{\boldsymbol{jk}}}({\boldsymbol{i}})}{{{\boldsymbol{\rho }}}_{{\boldsymbol{jk}}}}\,$$

where ρ_jk_ is the number of the shortest paths from node j to node k, and ρ_jk_(i) is the number of the shortest paths between node j and node k that pass through node i. Hence, BC(i) captures the influence of a node over information flow between other nodes in the network. Regions with a high betweenness centrality indicate high interconnectivity with other regions in the network.

The betweenness centrality map was calculated twenty times for each pre-defined individual connectivity matrix based on random network nodes at each scale using the Brain Connectivity Toolbox (http://www.brain-connectivity-toolbox.net). The individual betweenness centrality map was taken as an average of the twenty betweenness centrality maps^30^. We registered all individual betweenness centrality maps to a group template using a 2-dimensional surface-based registration algorithm^63, 64^. They were blurred with a 20 mm full width at half maximum surface-based diffusion kernel to decrease spatial variability between subjects^30^. This procedure was repeated for all nodal scales (Fig. 5(d)).

### Identifying H_IS_ regions

Each individual betweenness centrality map was normalized by subtracting the mean and divided by the standard deviation to allow unbiased comparison between the hub values of all the subjects. Averaged betweenness centrality map (*X*) at each scale was defined considering all individual betweenness centrality maps as:

2 $${\bf{X}}({\boldsymbol{i}})=\,\frac{{\bf{1}}}{{\boldsymbol{N}}}\sum _{{\boldsymbol{k}}={\bf{1}}}^{{\boldsymbol{N}}}\,{\boldsymbol{nBC}}({\boldsymbol{k}}),({\boldsymbol{i}}={\bf{1}}{\boldsymbol{,}}{\bf{2}}{\boldsymbol{\ldots }}{\boldsymbol{.}}{\boldsymbol{M}})$$

where *nBC(i)* is the normalized betweenness centrality map of individual *k*, *N* is the sets of all individuals, and *M* is the sets of scales. *X(i)* was calculated by averaging all normalized betweenness centrality maps across all individuals at each nodal scale. This equation was similar to a previous study^65^. H_IS_ strength (*H*_*IS*_ *_*_*ST*_) was defined considering all nodal scales as

3 $${{\boldsymbol{H}}}_{{\boldsymbol{IS}}\_{\boldsymbol{ST}}}({\boldsymbol{k}})=\frac{{\bf{1}}}{{\boldsymbol{M}}}\sum _{{\bf{i}}={\bf{1}}}^{{\boldsymbol{M}}}\,{\boldsymbol{X}}({\boldsymbol{i}}),({\boldsymbol{k}}={\bf{1}}{\boldsymbol{,}}{\bf{2}}{\boldsymbol{\ldots }}{\boldsymbol{.}}{\bf{V}})$$

where *X(i)* is the averaged betweenness centrality scores of scale *i*, *M* is the sets of scales, and *V* is the sets of vertices. *H*_*IS*_ *_*_*ST*_ *(k)* was defined as the sum of all normalized group hub scores divided by the total number of nodal scales in order to estimate the overall network hub pattern between multiple nodal scales. Note that the uncertainty of hub regions at the multiple nodal scales was averaged out^66–68^.

The *H*_*IS*_ score (*H*_*IS*_ *_*_*SC*_) was calculated to detect H_IS_ regions as:

4 $${{\boldsymbol{H}}}_{{\boldsymbol{IS}}\_{\boldsymbol{SC}}}({\boldsymbol{i}})=\frac{{{\boldsymbol{H}}}_{{\boldsymbol{IS}}\_{\boldsymbol{ST}}}({\boldsymbol{i}})-{\boldsymbol{\mu }}({{\boldsymbol{H}}}_{{\boldsymbol{IS}}\_{\boldsymbol{ST}}})}{{\boldsymbol{\sigma }}({{\boldsymbol{H}}}_{{\boldsymbol{IS}}\_{\boldsymbol{ST}}})}$$

where *i* is the node index of the *H*_*IS*_ *_*_*ST*_ map, ${\rm{\sigma }}$ denotes the standard deviation, and ${\rm{\mu }}$ denotes the mean. Z-score transformation was performed to combine the group hub scores from the different scales. The *H*_*IS*_ *_*_*SC*_ could capture how ‘well connected’ node i is to the other nodes in the *H*_*IS*_ *_*_*ST*_ map. Regions with high *H*_*IS*_ *_*_*SC*_ indicated a fundamental hub with high consistency across nodal scales. While most studies have suggested that z-scores of betweenness centrality could be used to identify the hub in that community at a single scale^38, 41^, *H*_*IS*_ regions were defined as regions with *H*_*IS*_ *_*_*SC*_ greater than a specific standard deviation, which was chosen as 1 in this study, plus the mean of the *H*_*IS*_ *_*_*ST*_ map.

### Validation analysis

A two-sample t-test was applied at multiple nodal scales to determine the statistical significance of the difference in local efficiency between the *H*_*IS*_ regions and the non *H*_*IS*_ regions at each nodal scale. The non *H*_*IS*_ region was defined as the network hub at each single scale that was not included in the *H*_*IS*_ regions. The network hubs tend to have a high level of local efficiency^36^. The local efficiency was defined as:

5 $${\boldsymbol{E}}({\boldsymbol{i}})=\,\sum _{{\boldsymbol{j}}\ne {\boldsymbol{i}}}^{{\boldsymbol{n}}}\frac{{{{\boldsymbol{d}}}^{-{\bf{1}}}}_{{\boldsymbol{ij}}}}{{\boldsymbol{n}}-{\bf{1}}}$$

where ${{{\boldsymbol{d}}}^{-1}}_{{\boldsymbol{ij}}}$ is the reciprocal of the shortest paths from node j to node i. We tested whether the *H*_*IS*_ regions showed a higher level of local efficiency than the non *H*_*IS*_ regions for each nodal scale. In addition, we used Kolmogorov–Smirnov normality test to check normality of their distribution using the SPSS Statistics 18 software (http://www.spss.com/software/statistics/).

### Data availability

Patient data was acquired from Samsung Medical Center and may not be made public due to restrictions from the IRB. Interested researchers may contact Dr. Sang Won Seo, a neurologist at SMC responsible for the data used in this study, to request access to confidential data^42–52, 59^.

## Electronic supplementary material

Supplementary information
