# Supplementary material for: Scale-integrated Network Hubs of the White Matter Structural Network
Source: Sci Rep. 2017 May 26;7:2449. doi: 10.1038/s41598-017-02342-7 (PMC5446418; doi:10.1038/s41598-017-02342-7)

**Scale-integrated Network Hubs of the White Matter Structural Network**

Hunki Kwon^1^, Yong-Ho Choi^1^, Sang Won Seo^2^, Jong-Min Lee^1*^

^1^Department of Biomedical Engineering, Hanyang University, Seoul, South Korea

^2^Department of Neurology, Samsung Medical Center, Sungkyunkwan University School of Medicine, Seoul, South Korea

***Corresponding author:**

Jong-Min Lee
Dept. of Biomedical Engineering, Hanyang University
Mailing Address: Sanhak-kisulkwan Room 319

222 Wangsipri-ro, Sungdong-gu, Seoul, KOREA, 133-791
Office: +82-2-2220-0685
E-mail: [ljm@hanyang.ac.kr](mailto:ljm@hanyang.ac.kr)

**Supplementary Table S1-S3**

**Supplementary Figure S1-S4**

**Supplementary Table S**1. The structural brain network hubs at various nodal scales.

| Study | | Modality | | Node scale | | Hub |  |
| --- | --- | --- | --- | --- | --- | --- | --- |
| Hagmann et al., 2008 | | DSI | | 998 | | Mid. Front; Mid. Pariet; Lateral Front Temp; Posterior Cingulat; Precun; Int Pariet; Int Front |  |
| Gong et al., 2009 | | DTI | | 78 | | Mid Occipit; Sup Occipit;  Precun; Sup Front;  Mid Sup Front |  |
| Zalesky et al., 2010 | | DTI,  HARDI | | 500, 1000 | | Cingulat |  |
|  |  |  |  | 3000 | | Ant Cingulat |  |
| Romero-Garcia et al.,  2012 | | Cortical  Thickness | | 66 | | Mid Front; Parahip; Cingulat;  Sup Temp |  |
|  |  |  |  | 108 | | Cuneus; Mid Occip; Mid Front; Precent; Cuneus; |  |
|  |  |  |  | 599 | | Inf Occipit; Sup Front;  Inf Occipit; Postcent; Inf Front;  Inf Front; Sup Temp. |  |
|  |  |  |  | 1494 | | Postcent; Mid Front; Precun;  Inf Pariet; Mid Front |  |
| H. J. Nijhuis et al.,  2013 | | DTI | | 500 | | Ant Sup Temp; Inf Pariet;  Post Temp; Ant Occipit |  |
|  |  | |  | |  | | |

DSI, diffusion spectrum imaging; DTI, diffusion tensor imaging; HARDI, high-angular resolution diffusion imaging; Post, posterior; Ant, anterior; Sup, superior; Mid, middle; Inf, inferior; Front, frontal; Parahippocamp, parahippocampal; Cingulat, cingulated; Temp, temporal; Occipit, occipital; Precent, precentral; Postcent, postcentral; Paracent, paracentral; Precun, precuneus; Pariet, parietal.

***Supplementary Table S2*** *Abbreviations for the cortical regions*

| Abgreviation | AAL Regions |
| --- | --- |
| PreCG.L | Left Precentral gyrus |
| PreCG.R | Right Precentral gyrus |
| SFGdor.L | Left Superior frontal gyrus, dorsolateral |
| SFGdor.R | Right Superior frontal gyrus, dorsolateral |
| ORBsup.L | Left Supeiror frontal gyrus, orbital part |
| ORBsup.R | Right Supeiror frontal gyrus, orbital part |
| MFG.L | Left Middle frontal gyrus |
| MFG.R | Right Middle frontal gyrus |
| ORBmid.L | Left Middle frontal gyrus orbital part |
| ORBmid.R | Right Middle frontal gyrus orbital part |
| IFGoperc.L | Left Inferior frontal gyrus, opercular part |
| IFGoperc.R | Right Inferior frontal gyrus, opercular part |
| IFGtriang.L | Left Inferior frontal gyrus, triangular part |
| IFGtriang.R | Right Inferior frontal gyrus, triangular part |
| ORBinf.L | Left Inferior frontal gyrus, orbital part |
| ORBinf.R | Right Inferior frontal gyrus, orbital part |
| ROL.L | Left Rolandic operculum |
| ROL.R | Right Rolandic operculum |
| SMA.L | Left Supplementary motor area |
| SMA.R | Right Supplementary motor area |
| OLF.L | Left Olfactory Cortex |
| OLF.R | Right Olfactory Cortex |
| SFGmed.L | Left Superior frontal gyrus, medial |
| SFGmed.R | Right Superior frontal gyrus, medial |
| ORBsupmed.L | Left Superior frontal gyrus, medial orbital |
| ORBsupmed.R | Right Superior frontal gyrus, medial orbital |
| REC.L | Left Gyrus Rectus |
| REC.R | Right Gyrus Rectus |
| INS.L | Left Insula |
| INS.R | Right Insula |
| ACG.L | Left Anterior cingulate and paracingulate gyri |
| ACG.R | Right Anterior cingulate and paracingulate gyri |
| DCG.L | Left Median cingulate and paracingulate gyri |
| DCG.R | Right Median cingulate and paracingulate gyri |
| PCG.L | Left Posterior cingulate gyrus |
| PCG.R | Right Posterior cingulate gyrus |
| PHG.L | Left Parahippocampal gyrus |
| PHG.R | Right Parahippocampal gyrus |
| CAL.L | Left Calcarine fissure and surrounding cortex |
| CAL.R | Right Calcarine fissure and surrounding cortex |
| CUN.L | Left Cuneus |
| CUN.R | Right Cuneus |
| LING.L | Left Lingual gyrus |
| LING.R | Right Lingual gyrus |
| SOG.L | Left Superior occipital gyrus |
| SOG.R | Right Superior occipital gyrus |
| MOG.L | Left Middle occipital gyrus |
| MOG.R | Right Middle occipital gyrus |
| IOG.L | Left Inferior occipital gyrus |
| IOG.R | Right Inferior occipital gyrus |
| FFG.L | Left Fusiform gyrus |
| FFG.R | Right Fusiform gyrus |
| PoCG.L | Left Postcentral gyrus |
| PoCG.R | Right Postcentral gyrus |
| SPG.L | Left Superior parietal gyrus |
| SPG.R | Right Superior parietal gyrus |
| IPL.L | Left Inferior parietal |
| IPL.R | Right Inferior parietal |
| SMG.L | Left Supramarginal gyrus |
| SMG.R | Right Supramarginal gyrus |
| ANG.L | Left Angular gyrus |
| ANG.R | Right Angular gyrus |
| PCUN.L | Left Precuneus |
| PCUN.R | Right Precuneus |
| PCL.L | Left Paracentral lobule |
| PCL.R | Right Paracentral lobule |
| HES.L | Left Heschl gyrus |
| HES.R | Right Heschl gyrus |
| STG.L | Left Superior temporal gyrus |
| STG.R | Right Superior temporal gyrus |
| TPOsup.L | Left Temporal pole: superior temporal gyrus |
| TPOsup.R | Right Temporal pole: superior temporal gyrus |
| MTG.L | Left Middle temporal gyrus |
| MTG.R | Right Middle temporal gyrus |
| TPOmid.L | Left Temporal pole: middle temporal gyrus |
| TPOmid.R | Right Temporal pole: middle temporal gyrus |
| ITG.L | Left Inferior temporal gyrus |
| ITG.R | Right Inferior temporal gyrus |

**Supplementary Table S3. Anatomical hub regions of the top 15 scores across different cortical scales.** Hubs were identified based on a normalized nodal betweenness centrality score, which was one standard deviation above the mean. Note that the ratio of the detected vertices to the whole vertices in the region of the pre-defined template was also given.

| **#nodal scale** | **100** | **200** | **300** | **400** | **500** | **600** |
| --- | --- | --- | --- | --- | --- | --- |
| **Hubs**  **(#vertices/ratio)** | PCUN.L(1915/84.4) | STG.L(953/62.2) | STG.L(972/63.4) | STG.L(988/64.5) | STG.L(1031/67.3) | STG.L(1027/67.0) |
|  | PCUN.R(1718/75.6) | ACG.R(929/86.3) | SPG.R(886/61.1) | SPG.R(933/64.4) | SPG.R(930/64.2) | SPG.R(931/64.2) |
|  | SPG.R(911/62.9) | CUN.L(813/62.1) | ACG.R(789/73.3) | ACG.R(685/63.6) | MTG.L(735/35.4) | MTG.L(692/33.3) |
|  | CUN.R(836/63.0) | SPG.R(786/54.2) | PCUN.L(723/31.8) | INS.L(645/61.9) | INS.L(653/62.6) | INS.L(650/62.3) |
|  | CUN.L(828/63.2) | PCUN.L(770/33.9) | PCUN.R(624/27.4) | PCUN.L(635/27.9) | PCUN.L(606/26.7) | ACG.R(564/52.4) |
|  | STG.L(732/47.8) | MTG.L(725/34.9) | CUN.L(622/47.5) | MTG.L(628/30.2) | ACG.R(592/55.0) | SOG.R(551/69.2) |
|  | SOG.R(632/79.3) | INS.L(641/61.5) | INS.L(579/55.5) | CUN.L(580/44.3) | SOG.R(562/70.6) | PreCG.L(543/45.5) |
|  | SPG.L(611/44.7) | SOG.R(623/78.2) | SOG.R(578/72.6) | SOG.R(562/70.6) | CUN.L(527/40.2) | PCUN.L(539/23.7) |
|  | INS.L(554/53.1) | CUN.R(610/46.0) | MTG.L(450/21.6) | PCUN.R(520/22.8) | PreCG.L(474/39.7) | CUN.L(476/36.3) |
|  | MTG.L(541/26.0) | PCUN.R(598/26.3) | CUN.R(430/32.4) | SFGdor.R(393/28.1) | SFGdor.R(421/30.2) | PreCG.R(465/39.3) |
|  | SOG.L(500/59.4) | SOG.L(533/63.3) | SFGdor.R(391/28.0) | CUN.R(392/29.5) | PCUN.R(415/18.2) | SFGdor.R(451/32.3) |
|  | PCL.L(440/52.2) | STG.R(415/23.1) | SOG.L(372/44.2) | INS.R(381/35.3) | SFGdor.L(347/21.7) | SFGdor.L(382/23.9) |
|  | MOG.R(350/25.4) | INS.R(372/34.5) | STG.R(361/20.1) | PreCG.L(364/30.5) | SMA.R(345/34.2) | SMA.R(356/35.3) |
|  | IFGtriang.L(229/29.2) | HES.L(271/1) | SFGdor.L(329/20.5) | SMA.R(341/33.8) | PreCG.R(334/28.2) | SMA.L(340/37.1) |
|  | IFGoperc.L(198/38.0) | SPG.L(263/19.2) | INS.R(325/30.1) | SFGdor.L(329/20.5) | CUN.R(331/24.9) | PCUN.R(308/13.5) |

**Supplementary Figure S1. Scale-integrated hub strength (*HIS_ST*).**

The hub strength was defined as the sum of all normalized group hub scores divided by the total number of nodal scales in order to estimate the overall network hub pattern between multiple nodal scales.


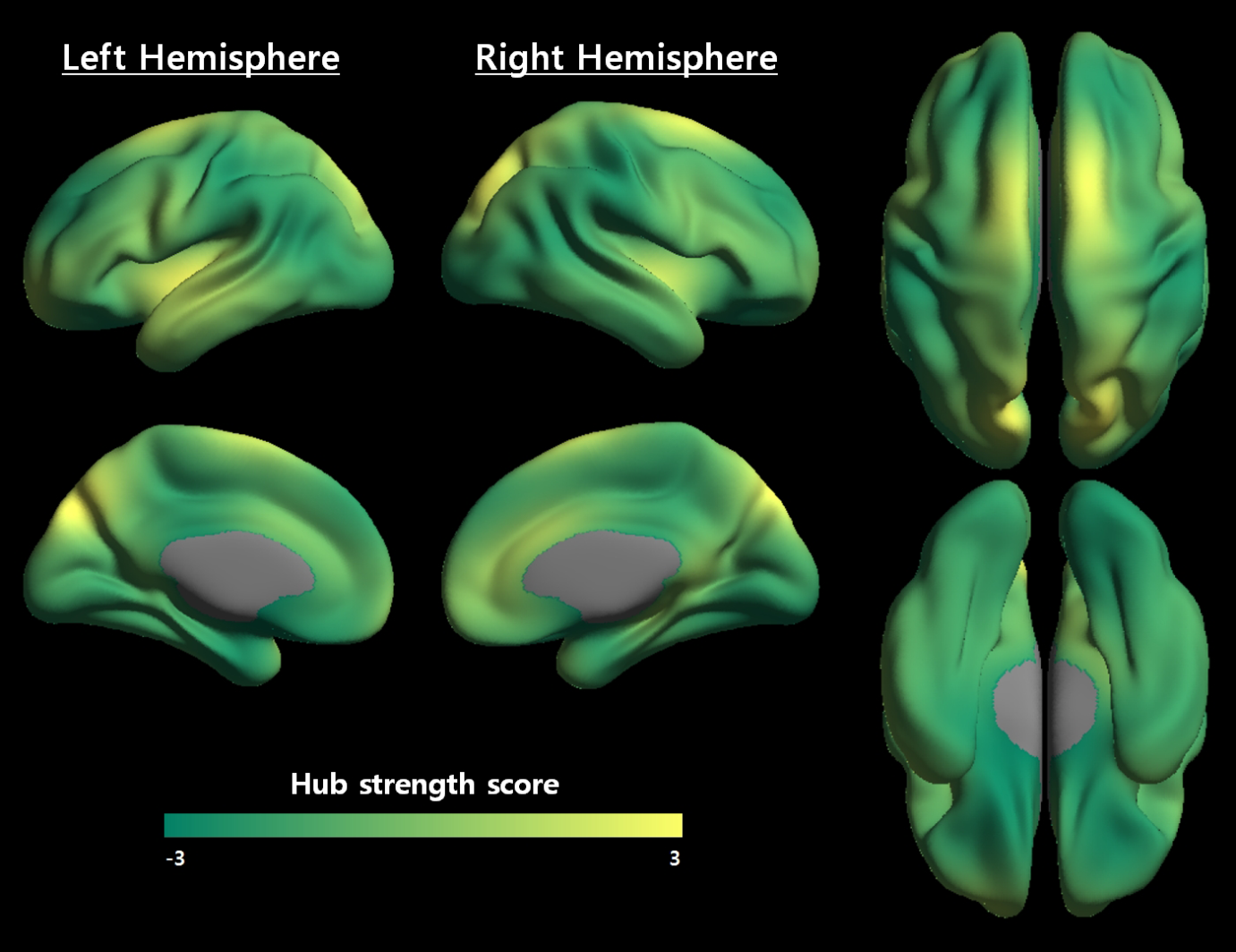


**Supplementary Figure S2. Distribution of betweenness centrality between the *H_IS_* regions and non *H_IS_* regions at each scale.**

The *H_IS_* regions had a tendency of increasing influences to betweenness centrality than the non *H_IS_* regions at most all scales. Note that the error bars represented the standard deviation.

**
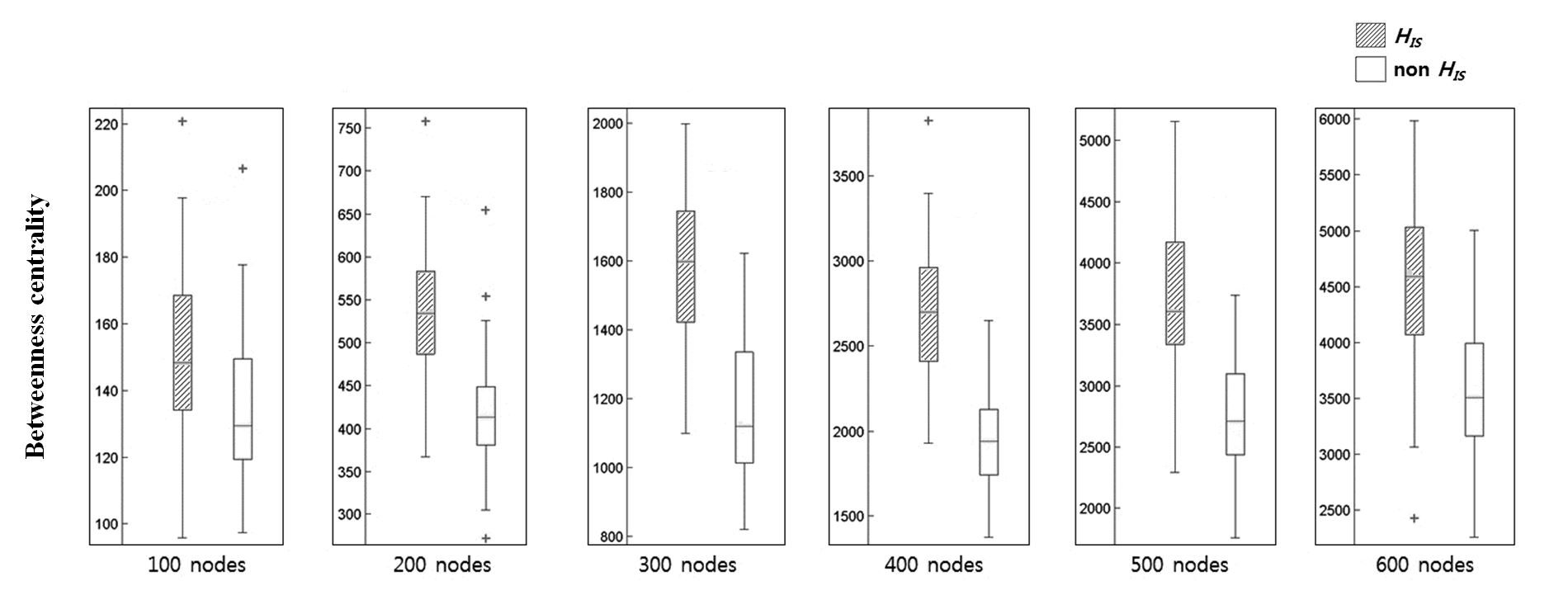
**

**Supplementary Figure S3. The number of network edges according to the threshold of fibers.**

The number of edges according to the threshold of fibers was calculated at each nodal scale. Various thresholds (1, 3, 5) were applied on the number of edges to see their effects at each nodal scale The number of edges at each nodal scale were averaged across all subjects.


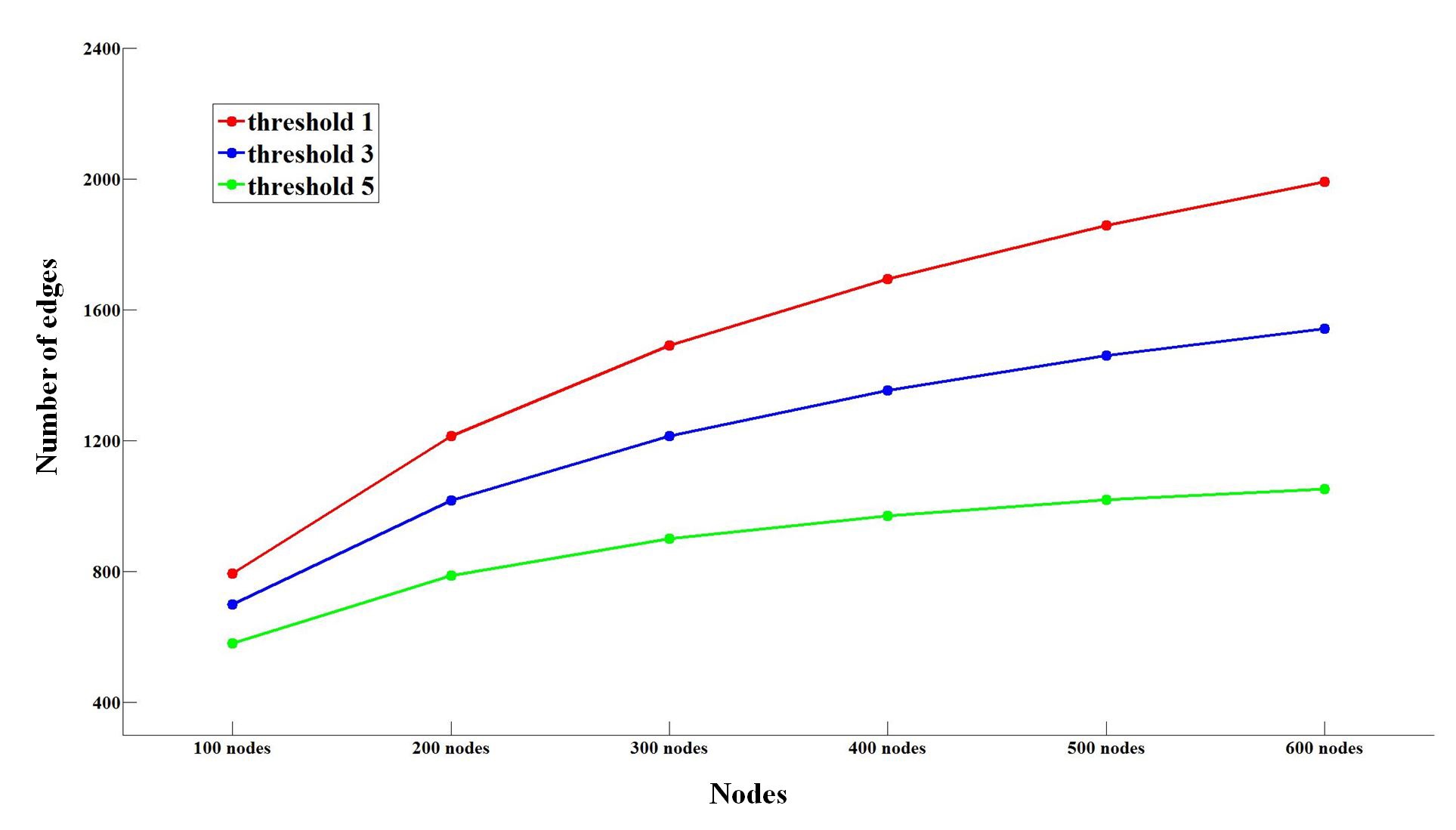


**Supplementary Figure S4. Distribution of scale-integrated hub (*H_IS_*) at multiple scale-integrated hub score (*HIS_SC*).**

Various HIS_SC (0.5, 1, 1.5, 2) values were applied to see their effects on determining the anatomical hub regions. The overall hub distribution showed a similar pattern even if the *HIS_SC* changed.


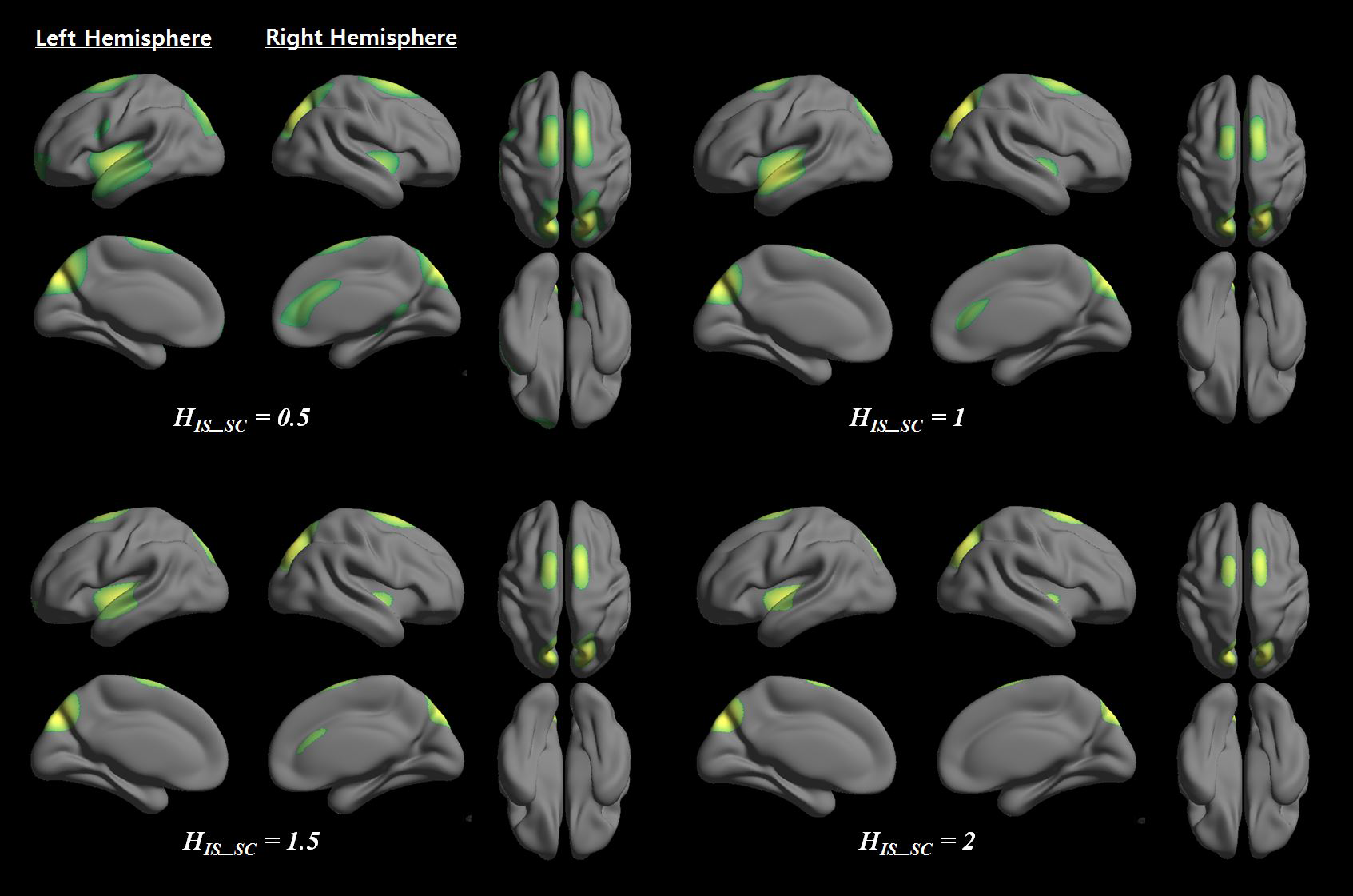

Supplement: Supplementary file 1 — Supplementary information [file 41598_2017_2342_MOESM1_ESM.doc]
